# Supplementary material for: Nutraceutical COMP-4 confers protection against endothelial dysfunction through the eNOS/iNOS-NO-cGMP pathway
Source: PLoS One. 2025 Feb 6;20(2):e0316798. doi: 10.1371/journal.pone.0316798 (PMC11801596; doi:10.1371/journal.pone.0316798)

		Area	Mean	Min	Max	adjusted
eNOS	blank	0.005	30	30	30	
	control	0.005	46.967	30	100	46.967
	IBMX	0.005	30.963	30	42	30.963
	control	0.005	57.046	30	119	57.046
	C4	0.005	124.68	34	255	124.680
	C4	0.005	126.824	34	255	126.824
	L-Arg	0.005	47.447	30	94	47.447
GAPDH	blank	0.005	30	30	30	
	control	0.005	89.695	36	189	59.695
	IBMX	0.005	120.134	42	255	90.134
	control	0.005	77.128	33	161	47.128
	C4	0.005	82.046	35	161	52.046
	C4	0.005	81.305	37	156	51.305
	L-Arg	0.005	82.78	34	186	52.780

30 ug protein loaded
eNOS 1:500
GEL Mini protean protean Gel 4-20%
Lycor photo documentation
1st membrane

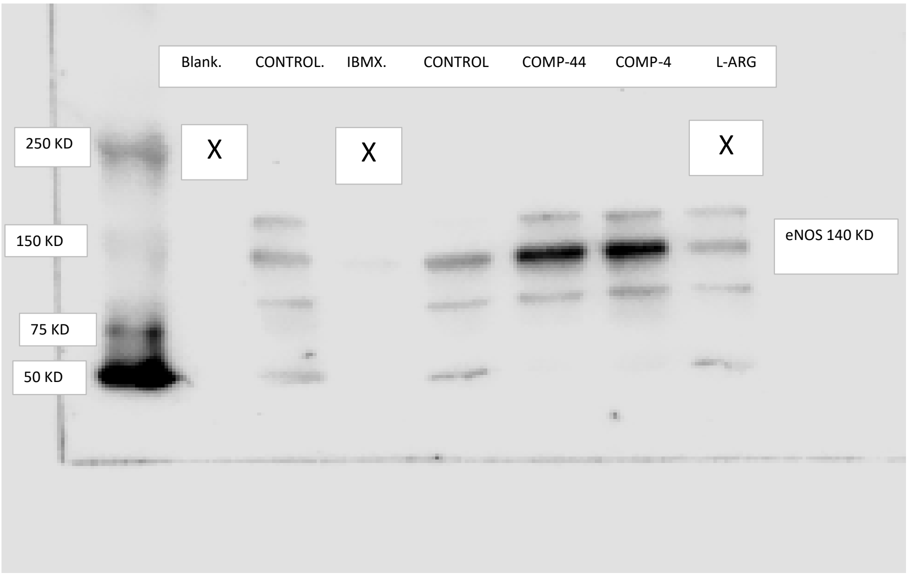

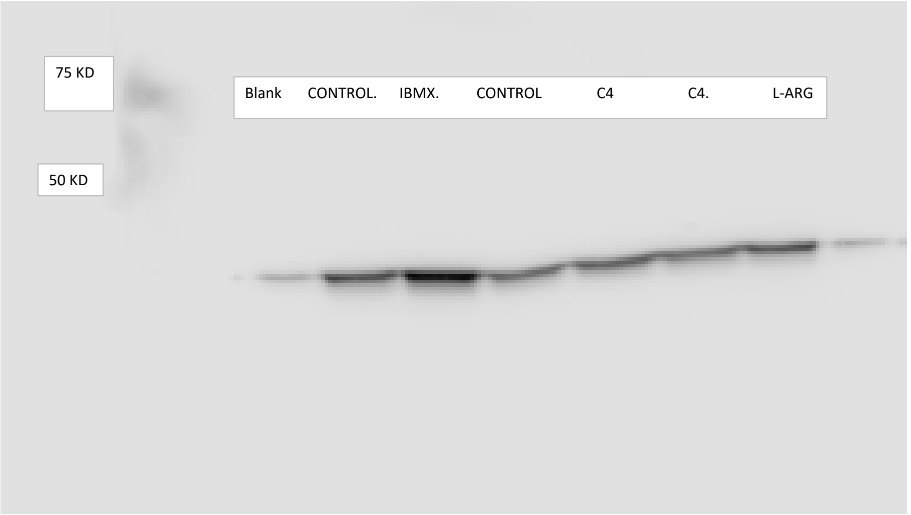

Supplement: S1 Fig — (PDF) [file pone.0316798.s001.pdf]
