# Supplementary material for: Nutraceutical COMP-4 confers protection against endothelial dysfunction through the eNOS/iNOS-NO-cGMP pathway
Source: PLoS One. 2025 Feb 6;20(2):e0316798. doi: 10.1371/journal.pone.0316798 (PMC11801596; doi:10.1371/journal.pone.0316798)

iNOS

		Area	Mean	Min	Max	adjusted
iNOS	blank	0.003	30	30	30	
	control	0.003	107.932	60	176	77.932
	IBMX	0.003	89.823	60	147	59.823
	control	0.003	96.295	60	156	66.295
	C4	0.003	139.885	61	215	109.885
	C4	0.003	153.386	63	232	123.386
	L-Arg	0.003	108.212	61	153	78.212
GAPDH	blank					
	control	0.008	141.507	43	255	39.468
	IBMX	0.008	158.161	62	255	56.122
	control	0.008	153.897	61	255	51.858
	C4	0.008	144.988	59	255	42.949
	C4	0.008	154.436	61	255	52.397
	L-Arg	0.008	137.095	55	255	35.056

30 ug protein loaded
iNOS 1:500
GEL Mini protean protean Gel 4-20%
Lycor photo documentation
1st membrane

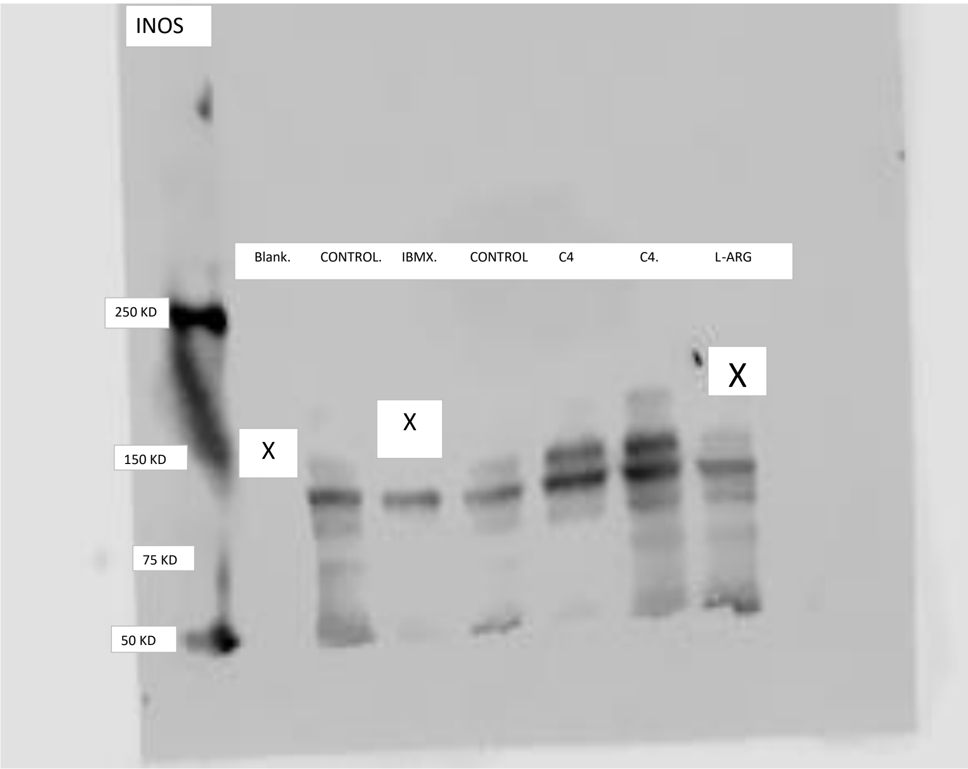

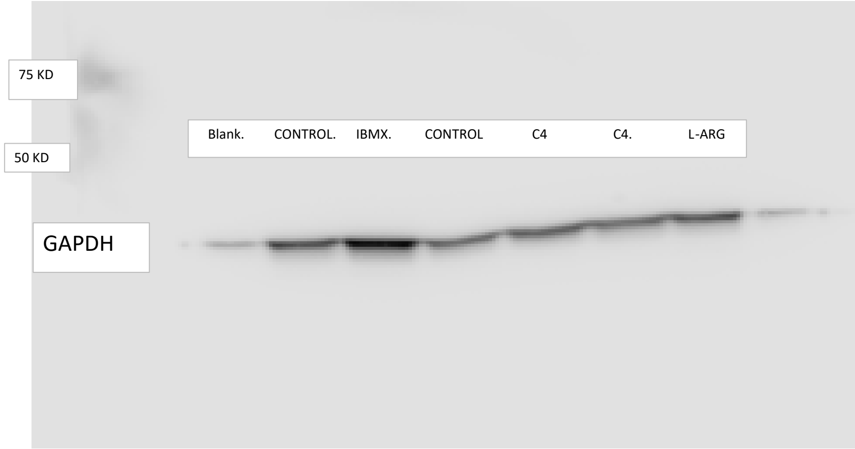

Supplement: S2 Fig — (PDF) [file pone.0316798.s002.pdf]
