# Supplementary material for: Nutraceutical COMP-4 confers protection against endothelial dysfunction through the eNOS/iNOS-NO-cGMP pathway
Source: PLoS One. 2025 Feb 6;20(2):e0316798. doi: 10.1371/journal.pone.0316798 (PMC11801596; doi:10.1371/journal.pone.0316798)
Supplement: S4 Fig — (PDF) [file pone.0316798.s004.pdf]

	Total Protein			
	Lane	Sample treatment	700 nm signal	Lane Normalization Factor
gel-1	1	Control	181	0.850
ptrotein	2	H202	153	0.718
12/10/21	3	C4	176	0.826
	4	C4+H2O2	208	0.977
	5	MP	213	1.000
gel-2	6	Control	65.7	0.877
medium	7	H202	69.6	0.929
12/10/21	8	C4	68	0.908
	9	C4+H2O2	70.5	0.941
	10	MP	74.9	1.000
10/28/21	11	Control	146	0.942
	12	PC	114	0.735
	13	MP	150	0.968
	14	Citr	155	1.000

	PAI (45 kDa)				
	Lane	Sample treatment	target signal	LNF (Revert)	Normalized to total protein
gel-1	1	Control	20.0	0.850	23.5
ptrotein	2	H202	23.3	0.718	32.4
12/10/21	3	C4	17.7	0.826	21.4
	4	C4+H2O2	20.4	0.977	20.9
	5	MP	19.2	1.000	19.2
gel-2	6	Control	27.2	0.877	31.0
medium	7	H202	27.2	0.929	29.3
12/10/21	8	C4	23.9	0.908	26.3
	9	C4+H2O2	26.6	0.941	28.3
	10	MP	23.8	1.000	23.8
10/28/21	11	Control	17.8	0.942	18.9
	12	PC	16.5	0.735	22.4
	13	MP	18.2	0.968	18.8
	14	Citr	17.1	1.000	17.1

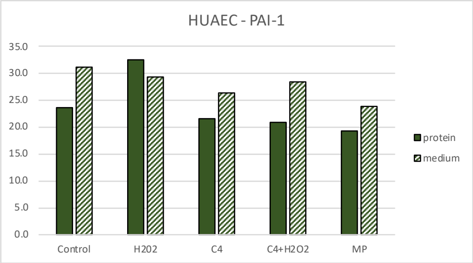

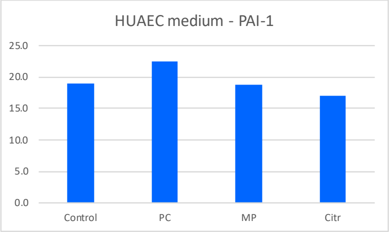

gel-1

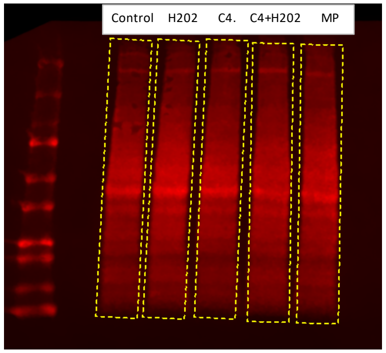

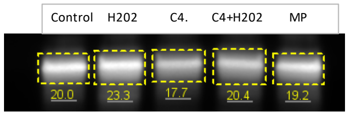

Fig 7

50 KD

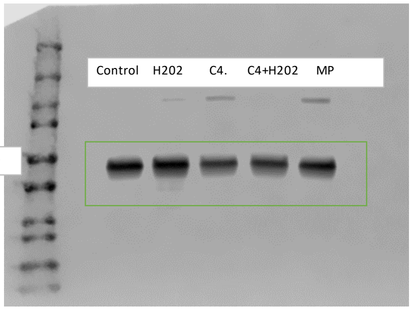

Gel 4-20%
Lycor photodcumentation
700 nm Signal

gel-2

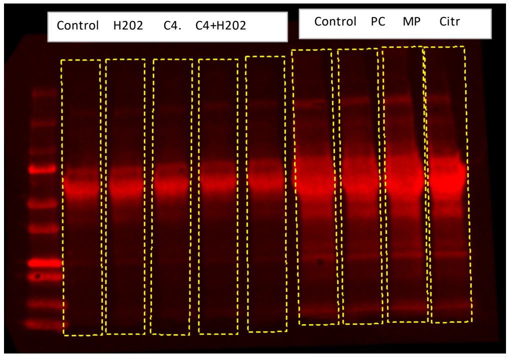

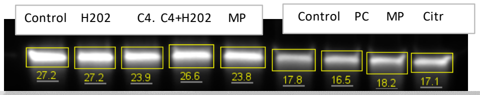

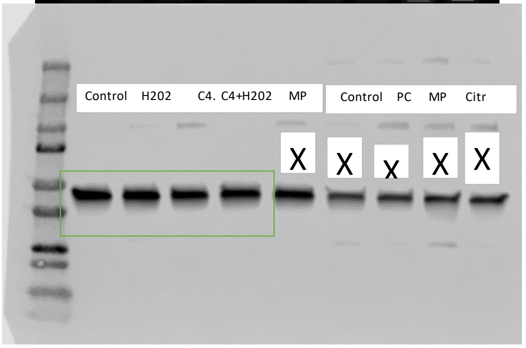

Fig 7
