# Supplementary material for: Nutraceutical COMP-4 confers protection against endothelial dysfunction through the eNOS/iNOS-NO-cGMP pathway
Source: PLoS One. 2025 Feb 6;20(2):e0316798. doi: 10.1371/journal.pone.0316798 (PMC11801596; doi:10.1371/journal.pone.0316798)
Supplement: S1 Table — (PDF) [file pone.0316798.s005.pdf]

	Group A	Group B	Group C	Group D	Group E	Group F	Group G	Group H
	Control	Sildenafil	IBMX	L-NAME	LNIL	COMP-4	COMP-4 + L-NAME	COMP-4 +L-NIL
1	11.82	75.6200	22.89	7.34	17.00	33.590	7.8600000	
2	18.13	37.5600	50.90	7.33	12.00	23.020	11.4300000	
3	9.38	37.4100	51.38	7.58	17.30	38.440	14.2067746	15.162
4	18.30	58.6000	25.33	9.68	16.25	32.680	19.7700000	19.770
5	18.00	51.2000	25.89	15.25		25.310		
6	13.89	31.4300	33.00			33.148		
7	18.90	21.2077	37.27					
