# Supplementary figures and images for: Nutraceutical COMP-4 confers protection against endothelial dysfunction through the eNOS/iNOS-NO-cGMP pathway
Source: PLoS One. 2025 Feb 6;20(2):e0316798. doi: 10.1371/journal.pone.0316798 (PMC11801596; doi:10.1371/journal.pone.0316798)

|                                                                                  |          |          |
|----------------------------------------------------------------------------------|----------|----------|
| 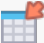 | Group A  | Group B  |
|                                                                                  | Control  | COMP-4   |
|                                                                                  |          |          |
| 1                                                                                | 116.0203 | 329.9807 |
| 2                                                                                | 109.9807 | 359.0000 |
| 3                                                                                | 93.0000  | 330.0000 |
| 4                                                                                |          | 440.0000 |

Supplement: S2 Table — (PDF) [file pone.0316798.s006.pdf]

| 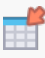 | Group A | Group B | Group C  | Group D    |
|----------------------------------------------------------------------------------|---------|---------|----------|------------|
|                                                                                  | Control | LPS     | NO DONOR | COMP-4     |
|                                                                                  |         |         |          |            |
| 1                                                                                | 36.00   | 64.000  | 65.00    | 55.0000000 |
| 2                                                                                | 41.50   | 47.388  | 68.00    | 52.0000000 |
| 3                                                                                | 47.00   | 67.000  | 68.50    | 51.0000000 |
| 4                                                                                | 42.28   | 79.000  | 41.41    | 67.5800000 |
| 5                                                                                | 30.97   | 69.000  |          | 67.0000000 |

Supplement: S3 Table — (PDF) [file pone.0316798.s007.pdf]

| 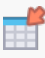 | Group A  | Group B  | Group C    | Group D  |
|----------------------------------------------------------------------------------|----------|----------|------------|----------|
|                                                                                  | Control  | IBMX     | SiLDENAFIL | COMP-4   |
|                                                                                  |          |          |            |          |
| 1                                                                                | 116.0203 | 121.0734 | 128.01     | 329.9807 |
| 2                                                                                | 109.9807 | 110.0000 | 130.00     | 359.0000 |
| 3                                                                                | 93.0000  | 78.3000  | 109.00     | 330.0000 |
| 4                                                                                |          |          |            | 440.0000 |

Supplement: S4 Table — (PDF) [file pone.0316798.s008.pdf]

| 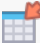 | Group A | Group B  | Group C |
|----------------------------------------------------------------------------------|---------|----------|---------|
|                                                                                  | Control | NO donor | COMP-4  |
|                                                                                  |         |          |         |
| 1                                                                                | 95.00   | 65.00    | 72.00   |
| 2                                                                                | 80.93   | 52.66    | 49.23   |
| 3                                                                                | 90.50   | 56.00    | 72.42   |
| 4                                                                                | 90.00   | 72.41    | 50.28   |

Supplement: S8 Table — (PDF) [file pone.0316798.s012.pdf]

| 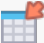 | Group A  | Group B  | Group C  | Group D     |
|----------------------------------------------------------------------------------|----------|----------|----------|-------------|
|                                                                                  | Control  | COMP-4   | H2O2     | H2O2+COMP-4 |
|                                                                                  |          |          |          |             |
| 1                                                                                | 190.0000 | 480.0000 | 110.0000 | 690.0000    |
| 2                                                                                | 216.0203 | 329.9807 | 178.0000 | 480.0000    |
| 3                                                                                | 238.0000 | 500.0000 | 140.0000 | 580.0000    |
| 4                                                                                | 250.9584 | 400.5284 | 205.1643 | 398.8145    |
| 5                                                                                |          |          |          | 539.5578    |

Supplement: S9 Table — (PDF) [file pone.0316798.s013.pdf]

| 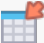 | Group A | Group B | Group C | Group D     |
|----------------------------------------------------------------------------------|---------|---------|---------|-------------|
|                                                                                  | CONTROL | COMP-4  | H2O2    | H2O2+COMP-4 |
|                                                                                  |         |         |         |             |
| 1                                                                                | 85.28   | 14.98   | 108.86  | 15.05       |
| 2                                                                                | 77.66   | 13.49   | 53.67   | 13.86       |

Supplement: S10 Table — (PDF) [file pone.0316798.s014.pdf]

| 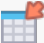 | Group A | Group B | Group C | Group D     |
|----------------------------------------------------------------------------------|---------|---------|---------|-------------|
|                                                                                  | CONTROL | COMP-4  | H2O2    | H2O2+COMP-4 |
|                                                                                  |         |         |         |             |
| 1                                                                                | 71.73   | 57.56   | 78.43   | 41.15       |
| 2                                                                                | 75.18   | 57.51   | 70.63   | 36.12       |

Supplement: S11 Table — (PDF) [file pone.0316798.s015.pdf]

| 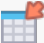 | Group A | Group B | Group C | Group D     |
|----------------------------------------------------------------------------------|---------|---------|---------|-------------|
|                                                                                  | CONTROL | COMP-4  | H2O2    | H2O2+COMP-4 |
|                                                                                  |         |         |         |             |
| 1                                                                                | 164.88  | 131.22  | 141.40  | 65.79       |
| 2                                                                                | 158.78  | 123.04  | 132.83  | 72.98       |

Supplement: S12 Table — (PDF) [file pone.0316798.s016.pdf]

| 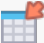 | Group A | Group B | Group C | Group D     |
|----------------------------------------------------------------------------------|---------|---------|---------|-------------|
|                                                                                  | CONTROL | COMP-4  | H2O2    | H2O2+COMP-4 |
|                                                                                  |         |         |         |             |
| 1                                                                                | 44.268  | 46.094  | 48.079  | 33.065      |
| 2                                                                                | 48.079  | 43.123  | 50.558  | 31.282      |

Supplement: S13 Table — (PDF) [file pone.0316798.s017.pdf]

|                                                                                  |         |         |         |             |
|----------------------------------------------------------------------------------|---------|---------|---------|-------------|
| 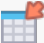 | Group A | Group B | Group C | Group D     |
|                                                                                  | CONTROL | COMP-4  | H2O2    | H2O2+COMP-4 |
|                                                                                  |         |         |         |             |
| 1                                                                                | 48.70   | 50.63   | 69.72   | 46.99       |
| 2                                                                                | 48.85   | 56.46   | 58.80   | 49.15       |

Supplement: S14 Table — (PDF) [file pone.0316798.s018.pdf]

| 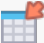 | Group A | Group B | Group C | Group D     |
|----------------------------------------------------------------------------------|---------|---------|---------|-------------|
|                                                                                  | Control | COMP-4  | H2O2    | H2O2+COMP-4 |
|                                                                                  |         |         |         |             |
| 1                                                                                | 16.21   | 10.99   | 20.44   | 6.63        |
| 2                                                                                | 16.54   | 11.41   | 16.65   | 11.70       |
| 3                                                                                | 13.21   | 11.24   | 17.81   | 12.92       |
| 4                                                                                | 18.04   | 10.59   | 14.28   | 13.08       |
| 5                                                                                |         | 12.34   | 17.23   |             |
| 6                                                                                |         |         | 24.56   |             |

Supplement: S15 Table — (PDF) [file pone.0316798.s019.pdf]

| 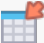 | Group A | Group B | Group C | Group D     |
|----------------------------------------------------------------------------------|---------|---------|---------|-------------|
|                                                                                  | CONTROL | COMP-4  | H2O2    | H2O2+COMP-4 |
|                                                                                  |         |         |         |             |
| 1                                                                                | 34.000  | 22.400  | 38.367  | 17.200      |
| 2                                                                                | 29.735  | 21.926  | 38.000  | 17.000      |
| 3                                                                                | 31.000  | 26.835  | 40.350  | 17.100      |
| 4                                                                                | 40.000  | 23.800  | 51.000  | 18.800      |
| 5                                                                                |         | 20.000  |         |             |

Supplement: S16 Table — (PDF) [file pone.0316798.s020.pdf]

| 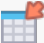 | Group A | Group B | Group C | Group D     |
|----------------------------------------------------------------------------------|---------|---------|---------|-------------|
|                                                                                  | Control | COMP-4  | H2O2    | H2O2+COMP-4 |
|                                                                                  |         |         |         |             |
| 1                                                                                | 16.21   | 10.99   | 20.44   | 6.63        |
| 2                                                                                | 16.54   | 11.41   | 16.65   | 11.70       |
| 3                                                                                | 13.21   | 11.24   | 17.81   | 12.92       |
| 4                                                                                | 18.04   | 10.59   | 14.28   | 13.08       |
| 5                                                                                |         | 12.34   | 17.23   |             |
| 6                                                                                |         |         | 24.56   |             |

Supplement: S17 Table — (PDF) [file pone.0316798.s021.pdf]
