# Supplementary material for: Nutraceutical COMP-4 confers protection against endothelial dysfunction through the eNOS/iNOS-NO-cGMP pathway
Source: PLoS One. 2025 Feb 6;20(2):e0316798. doi: 10.1371/journal.pone.0316798 (PMC11801596; doi:10.1371/journal.pone.0316798)
Supplement: S5 Table — (PDF) [file pone.0316798.s009.pdf]

Table format: Grouped		Group A nNOS					
		A:Y1	A:Y2	A:Y3	A:Y4	A:Y5	A:Y6
1	Control	1.000	1.000	1.000	1.000	1.000	
2	COMP-4	1.112	0.229	1.264	0.229	1.621	

		Group B eNOS					
		B:Y1	B:Y2	B:Y3	B:Y4	B:Y5	B:Y6
1	Control	1.000	1.000	1.000	1.000	1.000	1.000
2	COMP-4	4.530	3.191	5.542	4.187	7.389	

		Group C INOS					
		C:Y1	C:Y2	C:Y3	C:Y4	C:Y5	C:Y6
1	Control	1.000	1.000	1.000	1.000	1.000	
2	COMP-4	4.543	1.366	5.542	4.187	3.670	
