# Supplementary material for: Nutraceutical COMP-4 confers protection against endothelial dysfunction through the eNOS/iNOS-NO-cGMP pathway
Source: PLoS One. 2025 Feb 6;20(2):e0316798. doi: 10.1371/journal.pone.0316798 (PMC11801596; doi:10.1371/journal.pone.0316798)
Supplement: S6 Table — (PDF) [file pone.0316798.s010.pdf]

Table format: Grouped		Group A				Group B				Grou	
		nNOS				eNOS				INOS	
		A:Y1	A:Y2	A:Y3	A:Y4	B:Y1	B:Y2	B:Y3	B:Y4	C:Y1	C:Y2
1	CONTROL	1.00	1.00	1.00		1.00	1.00	1.00		1.00	1.00
2	COMP-4	0.36	0.36	0.26		6.40	6.80	3.46		2.09	2.28

Ta	up C	
	C:Y3	C:Y4
1	1.00	
2	3.20	
