# Supplementary material for: Nutraceutical COMP-4 confers protection against endothelial dysfunction through the eNOS/iNOS-NO-cGMP pathway
Source: PLoS One. 2025 Feb 6;20(2):e0316798. doi: 10.1371/journal.pone.0316798 (PMC11801596; doi:10.1371/journal.pone.0316798)
Supplement: S7 Table — (PDF) [file pone.0316798.s011.pdf]

Table format: Column		Group A	Group B	Group C
		Control	NO donor	COMP-4
1	Title	116.0	40	65.90
2	Title	127.5	51	27.50
3	Title	130.0	57	57.12
